# Whole-Brain Connectome Identifies PMv^LepRb^ Neurons as a Hypothalamic Hub Linking Metabolic State to Instinctive Behavior

**DOI:** 10.3390/cells15111027

**Published:** 2026-06-03

**Authors:** Xiang Zhang, Ye Dai, Yishuo Shi, Fang Yuan

**Affiliations:** 1 Department of Neurobiology, Hebei Medical University, Shijiazhuang 050017, China; zhangxiang@stu.hebmu.edu.cn (X.Z.); 24033100155@stu.hebmu.edu.cn (Y.D.); 15383710221@163.com (Y.S.); 2 Hebei Key Laboratory of Brain Science and Brain-Inspired Intelligence, Hebei Medical University, Shijiazhuang 050017, China; 3 The Key Laboratory of Neural and Vascular Biology, Ministry of Education, Hebei Medical University, Shijiazhuang 050017, China

**Keywords:** ventral premammillary nucleus, leptin receptor, monosynaptic connectomics, rabies virus, HSV

## Abstract

Metabolic state strongly shapes social and reproductive behaviors, yet the neural circuits that convert internal energy signals into behavioral responses remain poorly defined. The ventral premammillary nucleus (PMv) of the hypothalamus has been implicated in this process, particularly through leptin receptor-expressing (LepRb) neurons, but its brain-wide circuit organization is still unclear. Here, we used Cre-dependent retrograde (RV) and anterograde (HSV) viral tracing techniques in LepRb-Cre mice to construct a comprehensive, single-cell-resolution input–output map of PMv^LepRb^ neurons. 3D reconstruction showed that these neurons receive dense convergent inputs, mainly from hypothalamic and forebrain regions involved in energy balance, motivation, and limbic processing. In contrast, their outputs extend not only back to several input regions but also prominently to midbrain and pontine autonomic centers, including the periaqueductal gray (PAG) and parabrachial nucleus (PB). Quantitative analysis revealed that forebrain regions were more likely to participate in reciprocal connectivity, whereas brainstem regions were dominated by outgoing projections. This organization suggests that PMv^LepRb^ neurons are positioned to integrate metabolic and motivational signals and relay them to downstream systems controlling instinctive behavioral and autonomic responses. These findings provide a structural basis for understanding how energy state can influence decisions related to social competition and reproduction.

## 1. Introduction

Animals must continuously adjust their behavior in response to both internal physiological demands and changing environmental conditions. Factors such as hunger, energy reserves, social competition, and reproductive opportunities jointly shape behavioral decisions essential for survival [1,2]. In male animals, behaviors like territorial defense, dominance competition, and mate seeking are energetically costly and often carry significant risk. As a result, these behaviors must be tightly regulated according to the organism’s energetic state [3]. A long-standing concept in physiology is that the brain employs a neuroendocrine “gating” mechanism: when energy is limited, energetically expensive social and reproductive behaviors are suppressed; when energy is sufficient, these behaviors are permitted [4]. However, the neural basis of this gating process remains incompletely understood.

The hypothalamus plays a central role in coordinating homeostatic regulation and motivated behaviors, including feeding, thermoregulation, arousal, stress responses, and reproduction [5,6,7,8]. Within this network, the PMv has emerged as a key node linking metabolic signals to social and reproductive functions. Anatomical and functional studies have shown that the PMv is connected with multiple hypothalamic and limbic regions and is involved in pheromone processing, pubertal development, reproductive axis regulation, and male social behaviors [9,10]. However, the PMv contains heterogeneous neuronal populations, and its function cannot be fully understood at the level of the nucleus alone. Identifying the specific circuits of defined neuronal subtypes is therefore essential.

Leptin, an adipose-derived hormone, provides the brain with information about the body’s energy stores and is a critical regulator of energy homeostasis [11]. Disruption of leptin signaling—whether due to hormone deficiency or receptor dysfunction—leads to severe metabolic disturbances, including hyperphagia, obesity, and impaired reproductive function, often accompanied by altered social behaviors [12,13,14,15,16]. The PMv is one of the hypothalamic regions with the highest expression of the leptin receptor (LepRb), and a substantial proportion of its neurons—primarily Vglut2-positive excitatory neurons—are responsive to leptin [13,17], Previous studies have implicated PMv^LepRb^ neurons in pubertal initiation, gonadotropic regulation, and energy balance [14,18], suggesting that they may serve as a critical interface through which metabolic signals influence social and reproductive behaviors. Despite this, how PMv^LepRb^ neurons are embedded within brain-wide circuits remains largely unknown. Earlier anatomical studies provided only coarse descriptions of PMv connectivity and lacked the resolution to distinguish cell-type-specific input and output pathways [19,20]. More importantly, it remains unclear how PMv^LepRb^ neurons integrate signals from diverse upstream regions and relay them to downstream structures that ultimately drive behavior. Addressing this question requires a comprehensive, cell-type-specific mapping of both afferent and efferent connections. Recent advances in viral tracing techniques have made such analyses possible. Modified RV allows for the identification of direct monosynaptic inputs to genetically defined neurons [21], while HSV-based anterograde tracing enables mapping of their downstream projection targets [22,23]. Together, these approaches provide a powerful strategy for reconstructing complete input–output connectivity at the whole-brain level.

In this study, we combined Cre-dependent RV and HSV tracing in LepRb-Cre mice to map the brain-wide input and output architecture of PMv^LepRb^ neurons. We aimed to identify the major upstream sources conveying metabolic and social information to the PMv, as well as the downstream targets that may mediate behavioral and physiological responses. In addition, we asked whether the connectivity of PMv^LepRb^ neurons follows an underlying organizational principle that could explain how internal energy states are transformed into adaptive behaviors. By establishing a comprehensive connectome of PMv^LepRb^ neurons, this work provides a structural framework for understanding how metabolic signals interact with social and environmental information to shape behavior and offers new insights into the neural basis of disorders involving metabolic imbalance, reproductive dysfunction, and altered social behavior.

## 2. Materials and Methods

### 2.1. Animals

A total of 16 male *LepRb-Cre* mice (stock #008320; The Jackson Laboratory, Bar Harbor, ME, USA) were initially utilized for the viral tracing experiments, with 8 mice allocated to retrograde monosynaptic (RV) tracing and 8 mice allocated to anterograde monosynaptic (HSV) tracing. All experimental protocols were approved by the Animal Care and Ethical Committee of Hebei Medical University (Approval No. Hebmu-2019001) and conducted in strict accordance with the Guide for the Care and Use of Laboratory Animals. Mice used in this study were aged between postnatal days 56 and 84 (P56–P84). Animals were maintained under a standard 12 h light/dark cycle (lights on at 07:00 UTC+8) with ad libitum access to food and water. At the conclusion of the experiments, all mice were humanely euthanized via an overdose of sodium pentobarbital (>100 mg/kg). All viral tracing experiments were conducted within designated Biosafety Level 2 (BSL-2) laboratory and animal housing facilities.

### 2.2. Viral Vectors and Surgery

All packaged viral vectors were purchased from BrainCase (Braincase, Shenzhen, China) and stored at −80 °C until use. All AAV helper vectors utilized in this study were of the AAV2/9 serotype. For retrograde monosynaptic tracing, the rabies virus (RV-EnvA-ΔG-mCherry) was used at a titer of ~2 × 10^8^ IFU/mL. The matched Cre-dependent AAV helpers included AAV-EF1α-DIO-RG (expressing RV-G; 3.15 × 10^12^ vg/mL) and AAV-EF1α-DIO-TVA-EGFP (expressing the TVA receptor; 3.05 × 10^12^ vg/mL). For strict anterograde monosynaptic tracing, the herpes simplex virus (H.129-ΔgD-hUbC-EGFP-scHer2::gD) was applied at a titer of ~1 × 10^8^ IFU/mL. The corresponding Cre-dependent AAV helpers were AAV-UL26.5-DIO-cmgD (expressing cmgD; 3 × 10^12^ vg/mL) and AAV-hSyn-DIO-mCherry-Her2CT9 (expressing the Her2 receptor; 3.2 × 10^12^ vg/mL) [23].

Stereotaxic surgeries were performed using a stereotaxic instrument (RWD Life Science, Shenzhen, China) under continuous 2% isoflurane anesthesia, with body temperature stabilized at 37 °C via a feedback-controlled heating pad and eyes protected by carbomer ointment. AAV helper cocktails for retrograde (AAV-EF1α-DIO-TVA-EGFP and AAV-EF1α-DIO-RG, 1:2 ratio) or anterograde (AAV-hSyn-DIO-mCherry-Her2CT9 and AAV-UL26.5-DIO-cmgD, 1:2 ratio) tracing were prepared prior to surgery. A volume of 50 nL of the selected AAV mixture was delivered unilaterally into the PMv (AP: −2.35 mm, ML: −0.50 mm, DV: −5.95 mm) [24] at 20 nL/min. The glass electrode was maintained at the injection site for 10 min to facilitate viral diffusion and minimize backflow. Following a two-week incubation, a secondary injection of either RV-EnvA-ΔG-mCherry (50 nL) or H.129-ΔgD-hUbC-EGFP-scHer2::gD (50 nL) was targeted to the same coordinates. The skull defect was then sealed with bone cement, and the skin was sutured following thorough disinfection. Mice were administered subcutaneous carprofen (5 mg/kg) and allowed to recover fully under thermal support. Following a 7-day post-operative monitoring period assessing weight, behavior, and feeding/drinking activity, animals were subjected to transcardial perfusion for immunohistological evaluation.

### 2.3. Histology

One week post-RV/HSV injection, mice were deeply anesthetized (sodium pentobarbital, 50 mg/kg, i.p.) and transcardially perfused with saline, followed by ice-cold 4% PFA in 0.1 M PB (pH 7.4). Brains were harvested, post-fixed overnight (12–16 h) in 4% PFA at 4 °C, and dehydrated in a 10–30% graded sucrose gradient until saturated. Following embedding in OCT compound and storage at −80 °C, 25-μm coronal sections were collected in four series using a cryostat (CM1950, Leica, Wetzlar, Germany). For immunolabeling, sections were blocked and permeabilized in 0.01 M PBS containing 5% BSA and 0.25% Triton X-100 and then incubated for 12 h at 4 °C with primary antibodies: chicken anti-EGFP (1:1000; Abcam, Cambridge, UK, ab13970) and rabbit anti-mCherry (1:1000; Abcam, ab167453). After thorough washing in PBS, sections were incubated with Alexa Fluor^®^ 488-conjugated goat anti-chicken (1:1000; Abcam, ab150169) and Alexa Fluor^®^ 555-conjugated donkey anti-rabbit (1:1000; Abcam, ab150074) secondary antibodies for 2 h at room temperature in the dark. Sections were subjected to three final PBS washes and coverslipped with Fluoromount-G^®^ (SouthernBiotech, Birmingham, AL, USA).

For whole-brain spatial mapping, consecutive coronal sections were sequentially mounted on glass slides along the anterior-to-posterior axis, maintaining a 100 μm sampling interval. Whole-slide image acquisition and subsequent stitching were performed using a fluorescence microscope (Axio Imager 2, Carl Zeiss, Jena, Germany).

### 2.4. Image Analysis and Spatial Registration

To ensure high anatomical precision and reliability of the connectome map, strict post hoc histological exclusion criteria were applied. Samples were excluded from subsequent whole-brain analysis if they exhibited viral leakage or spillover outside the anatomical boundaries of the PMv or if the expression of starter neurons was too low to be reliably observed. Based on these criteria, 6 mice were excluded due to off-target expression or poor viral transduction. Consequently, a final analyzed cohort of 10 mice (5 for RV tracing and 5 for HSV tracing) successfully met the quality control standards and was included in the final analyses. To reconstruct the global distribution of virally labeled neurons, we utilized an open-source image analysis pipeline. Raw fluorescence images were preprocessed in QuPath (v0.5.1) [25] and subjected to automated somatic segmentation using the Cellpose plugin (cyto3 model) [26]. The segmentation accuracy was further optimized through a “human-in-the-loop” training paradigm [27] and validated via exhaustive manual slice-by-slice correction. Sub-optimally registered slices were systematically adjusted using the BigWarp plugin within Fiji (v2.17.0). This manual fine-tuning was primarily guided by local neuroanatomical landmarks, including the shape of the third ventricle, the fornix, and the mammillothalamic tract.

To map these neurons into a standardized neuroanatomical space, the acquired sections were nonlinearly and elastically registered to the Allen CCFv3 using the ABBA plugin [28,29], yielding absolute 3D coordinates for all targeted cells. These coordinates were imported into a Python (v3.7.0) environment, where the brainrender library [30] was used to construct whole-brain point clouds of the projection networks. For advanced rendering, regional meshes and point clouds were exported as OBJ models via vedo and visualized using Blender (v4.4; Blender Foundation, Amsterdam, The Netherlands).

For brain-wide quantification, labeled neurons were automatically assigned to specific neuro-anatomical structures based on CCFv3 parcellations, generating absolute cell counts for statistical analysis. Additionally, to resolve the fine-scale anterior–posterior topographical gradients of projection neurons within target nuclei, coronal cell counts were computed and visualized as 2D spatial heatmaps using the brainglobe-heatmap package [31].

### 2.5. Statistical Analysis and Data Visualization

Regional connection strengths were normalized as a fraction of total input or output neurons and subsequently log-transformed via log_10_(x + 1). To facilitate a direct, scale-independent comparison of afferent and efferent projections, these transformed data were standardized into Z-scores.

Brain regions were then segregated into four connectivity modules based on strictly defined Z-score thresholds: High-In/High-Out (Z_in_ ≥ 0.8, Z_out_ ≥ 0.8), High-In/Low-Out (Z_in_ ≥ 0.8, 0 ≤ Z_out_ < 0.8), Low-In/High-Out (0 ≤ Z_in_ < 0.8, Z_out_ ≥ 0.8), and Low-In/Low-Out (Z_in_ < 0, Z_out_ < 0). To assess input–output dominance within each module, standardized Z-scores were statistically compared using Mann–Whitney U tests via GraphPad Prism (v9.0; GraphPad Software, San Diego, CA, USA). Results were visualized as violin plots overlaid with individual data points. Significance thresholds were set at *p* < 0.05 (denoted as ** *p* < 0.01, **** *p* < 0.0001, and n.s. = not significant).

Furthermore, to elucidate the topological similarity of projection patterns, agglomerative hierarchical clustering was applied to the regions within each module using their individual log_10_(x + 1) values. Utilizing the Euclidean distance metric and the average linkage method, a hierarchical dendrogram was generated. This clustering hierarchy dictated the final sorting of the connectivity matrix, which was visualized as a high-contrast heatmap.

## 3. Results

### 3.1. Viral Tracing Strategies for Mapping the Brain-Wide Inputs and Outputs of PMv^LepRb^ Neurons

**Figure 1:**
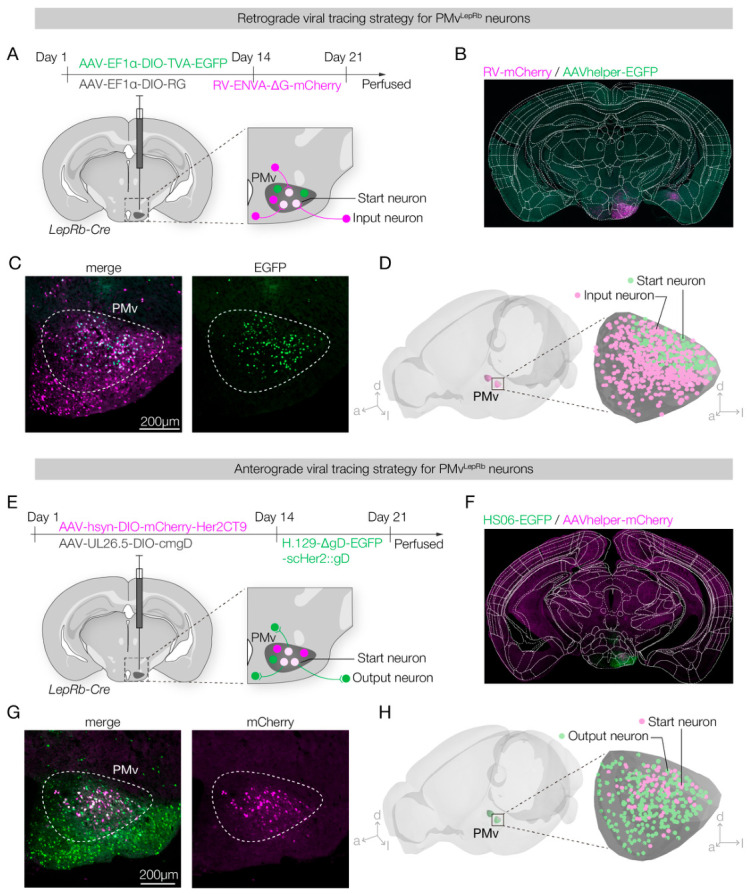
Validation of monosynaptic retrograde and anterograde tracing in PMv^LepRb^ neurons: (**A**) Schematic of the Cre-dependent retrograde rabies virus (RV) tracing strategy in LepRb-Cre mice. Timeline shows the sequential PMv injection of helper AAVs (Day 1) and RV (Day 14), followed by perfusion (Day 21). (**B**,**C**) Representative images at the PMv injection site. Targeted infection is confirmed by the specific colocalization of helper AAVs (EGFP, green) and RV (mCherry, magenta). (**D**) 3D spatial mapping illustrating the topographical distribution of starter neurons (green) and upstream neurons (magenta) within the PMv anatomical outline (gray). (**E**) Schematic of the anterograde HSV tracing strategy, with sequential injection of helper AAVs (Day 1) and recombinant HSV (Day 14). Perfusion was performed on Day 21. (**F**,**G**) Representative images showing specific colocalization of helper AAVs (mCherry, magenta) and HSV (EGFP, green) at the PMv injection site. (**H**) 3D spatial mapping of starter neurons (magenta) and downstream neurons (green) within the PMv.

To systematically dissect the whole-brain afferent and efferent connectivity of leptin receptor-expressing neurons in the PMv, we employed highly specific, Cre-dependent monosynaptic viral tracing strategies. For the retrograde mapping of presynaptic inputs, a modified rabies virus system was utilized. We first stereotaxically injected a helper AAV cocktail containing AAV-EF1α-DIO-TVA-EGFP and AAV-EF1α-DIO-RG into the PMv of LepRb-Cre mice. Following a two-week integration period, the RV-EnvA-ΔG-mCherry was delivered to the same coordinates (Figure 1A). This intersectional strategy ensured that the TVA receptor and rabies glycoprotein were exclusively expressed in PMv^LepRb^ neurons, enabling the RV to specifically infect these cells and retrogradely traverse across a single synapse. Histological verification revealed highly restricted expression of the helper AAVs (EGFP-positive) and successful RV infection (mCherry-positive) localized within the PMv (Figure 1B,C). Within this region, EGFP/mCherry double-labeled cells were identified as the “starter neurons” from which the retrograde tracing originated, whereas cells expressing exclusively mCherry represented the monosynaptic input neurons (Figure 1D).

Complementarily, to delineate the direct postsynaptic targets of PMv^LepRb^ neurons, we implemented a strict anterograde monosynaptic tracing approach utilizing the H129 strain of HSV [23]. A mixture of helper AAVs (AAV-hSyn-DIO-mCherry-Her2CT9 and AAV-UL26.5-DIO-cmgD) was injected into the PMv, followed 14 days later by the delivery of the recombinant HSV (H129ΔgD-EGFP-scHer2::gD) (Figure 1E). High-resolution imaging confirmed the precise colocalization of the helper viruses (mCherry-positive) and the anterograde HSV (EGFP-positive) at the injection site (Figure 1F,G). Correspondingly, mCherry/EGFP double-positive cells were designated as the anterograde starter neurons, while EGFP-only cells indicated the trans-synaptically labeled output neurons (Figure 1H). High-fidelity 3D spatial reconstructions further corroborated the discrete topographical distribution of these defined starter populations and their local network architecture within the PMv (Figure 1D,H), thereby robustly validating the accuracy and reliability of both viral pipelines for subsequent brain-wide connectivity analyses. High-resolution 3D spatial reconstructions provided clear evidence for the distribution patterns of the defined starter neurons and their trans-synaptically labeled pre- and postsynaptic partners within the PMv (Figure 1D,H). Ultimately, these data validate the precision and robustness of the dual viral tracing systems, enabling highly reliable mapping of brain-wide connectivity networks in subsequent analyses.

### 3.2. Global Architecture of the PMv^LepRb^ Afferent and Efferent Networks

**Figure 2:**
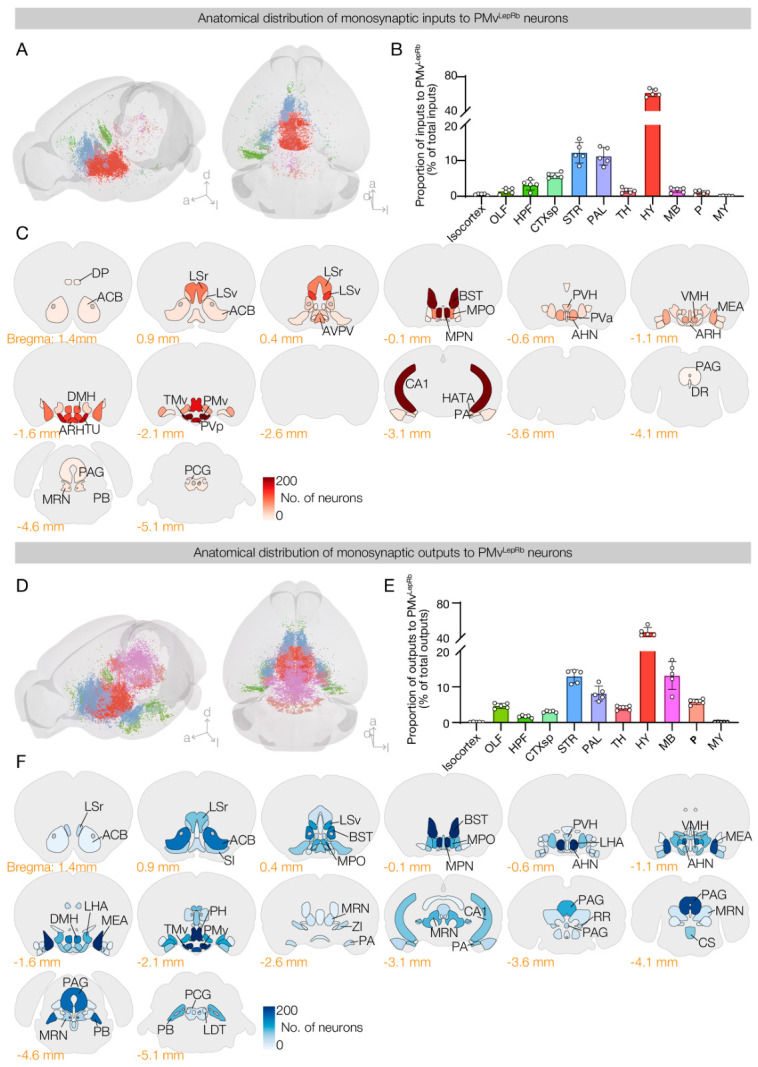
Brain-wide anatomical mapping of PMv^LepRb^ monosynaptic connectomes: (**A**–**C**) Brain-wide distribution of upstream inputs. (**A**) 3D spatial reconstructions (sagittal and horizontal views) showing the topographical distribution of the afferent network. (**B**) Proportional quantification of inputs across major brain divisions (mean ± SEM, *n* = 5 mice). (**C**) 2D coronal maps along the A-P axis (Bregma 1.4 mm to −5.1 mm), with heatmap gradients indicating absolute cell counts within specific nuclei. (**D**–**F**) Brain-wide distribution of downstream outputs. (**D**) 3D spatial reconstruction of monosynaptic efferent targets. (**E**) Proportional quantification of outputs across major brain divisions (mean ± SEM, *n* = 5 mice). (**F**) Representative 2D coronal maps of output neurons, with heatmap gradients representing absolute cell counts in target nuclei. For a complete definition of all neuroanatomical abbreviations, readers are referred to the Allen Mouse Brain Common Coordinate Framework (CCFv3) interactive atlas available at: https://atlas.brain-map.org/, accessed on 20 December 2025.

To elucidate the upstream and downstream neural networks monosynaptically connected to PMv^LepRb^ neurons, we analyzed the brain-wide distribution of RV-mCherry-labeled trans-synaptic input neurons and HSV-EGFP-labeled output neurons across serial coronal sections from five mice per viral group. 3D spatial mapping revealed that both afferent and efferent neurons exhibited a widespread yet highly organized distribution along the anterior–posterior axis (Figure 2A,D).

**Figure 3:**
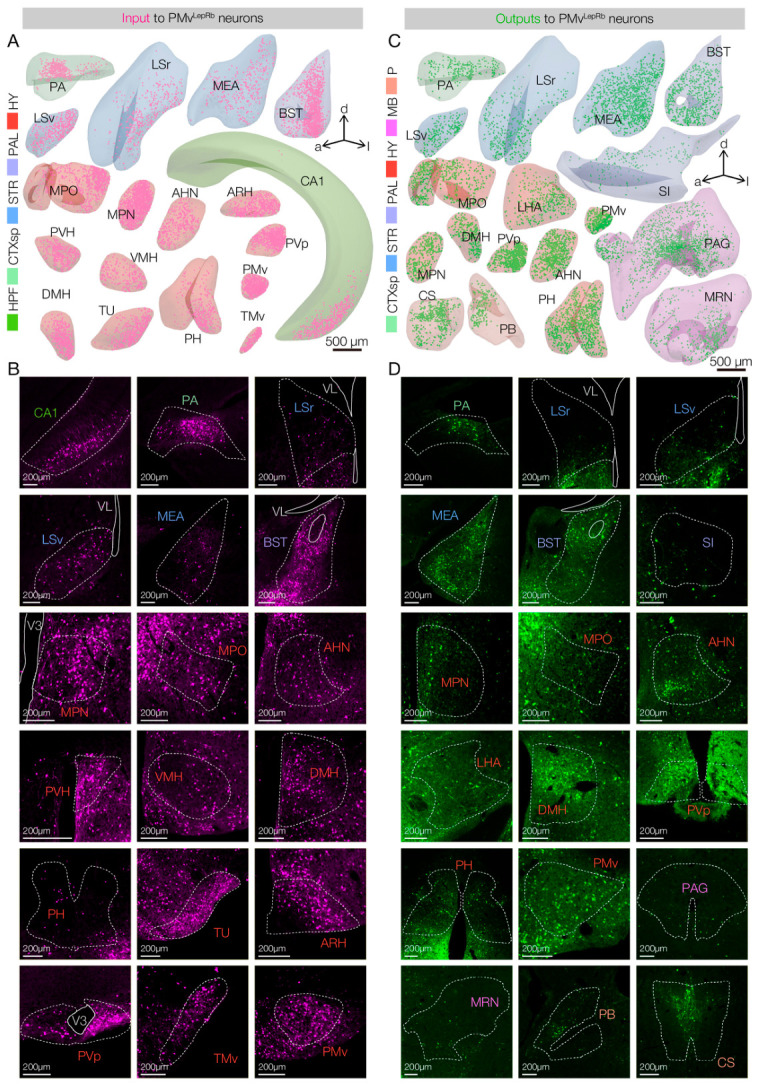
The 3D spatial reconstruction and morphological validation of PMv^LepRb^ major input and output nuclei: (**A**) High-resolution 3D regional modeling of core upstream inputs. Translucent outlines delineate the anatomical boundaries of representative nuclei, with internal magenta spheres mapping the exact spatial topography of mCherry-positive retrogradely labeled neurons. The left color bar indicates the primary anatomical brain divisions. (**B**) Representative fluorescence images of major input nuclei. White dashed lines outline regional boundaries to highlight the dense clustering of magenta input somata. Scale bars as indicated. (**C**) High-resolution 3D regional modeling of core downstream targets. Green spheres indicate the spatial topography of EGFP-positive anterogradely labeled neurons and terminal arborizations. (**D**) Representative images of major output targets. Green fluorescence explicitly visualizes the distribution of downstream targets. Scale bars as indicated.

Quantitative analysis of major anatomical divisions demonstrated that PMv^LepRb^ neurons receive their predominant afferent innervation from the hypothalamus (HY), accounting for 62.13% of total brain-wide inputs. Secondary input sources originated from the striatum (STR; 11.77%), pallidum (PAL; 11.12%), and cortical subplate (CTXsp; 5.7%) (Figure 2B). Coronal heatmaps and 3D nuclear reconstructions were utilized to further resolve the spatial expression of these upstream neurons within specific brain nuclei (Figure 2C and Figure 3A). Within the hypothalamus, input neurons were densely clustered in the MPO, MPN, VMH, and ARH. Notably, robust extra-hypothalamic inputs primarily emanated from the BST in the PAL, the LSr, LSv, and MEA in the STR, alongside a prominent projection from the hippocampal CA1 region. In total, 176 brain regions were identified as providing afferent projections. To intuitively visualize the primary distribution characteristics of mCherry-labeled presynaptic neurons, we presented representative high-magnification images of the top 18 nuclei with the highest neuron counts (Figure 3B).

To map the downstream target regions, we employed the HSV-based anterograde tracing system. Although the efferent networks spatially overlapped with the afferent architecture, the projection targets exhibited striking divergence (Figure 2D). Brain-wide quantification revealed that the hypothalamus remained the primary target, constituting 45.28% of total outputs. However, in stark contrast to the input patterns, PMv^LepRb^ neurons sent dense descending projections to the midbrain (MB; 13.58%) and pons (P; 5.9%)—regions that provided negligible reciprocal inputs (Figure 2E).

At the nucleus level, efferent density heatmaps and localized 3D models indicated that axon terminals intricately arborized within key hypothalamic structures, including the LHA, AHN, and DMH (Figure 2F and Figure 3C). Extra-hypothalamic forebrain targets prominently featured the BST, MEA, and lateral septum (LSr, LSv). Diverging from the retrograde tracing results, PMv^LepRb^ neurons densely innervated multiple hindbrain nuclei, particularly the PAG, MRN, and PB (Figure 2F and Figure 3C). High-resolution magnified images of EGFP-positive neurons structurally validated these critical downstream anatomical nodes (Figure 3D). These anatomical tracing data reveal a highly asymmetrical connectome: PMv^LepRb^ neurons integrate dominant inputs from the forebrain and hypothalamus and concurrently form dense local reciprocal connections, subsequently relaying diverse signals to long-range autonomic centers in the midbrain and hindbrain.

### 3.3. Quantitative Profiling of Brain-Wide Afferent and Efferent Nuclei of PMv^LepRb^ Neurons

**Figure 4:**
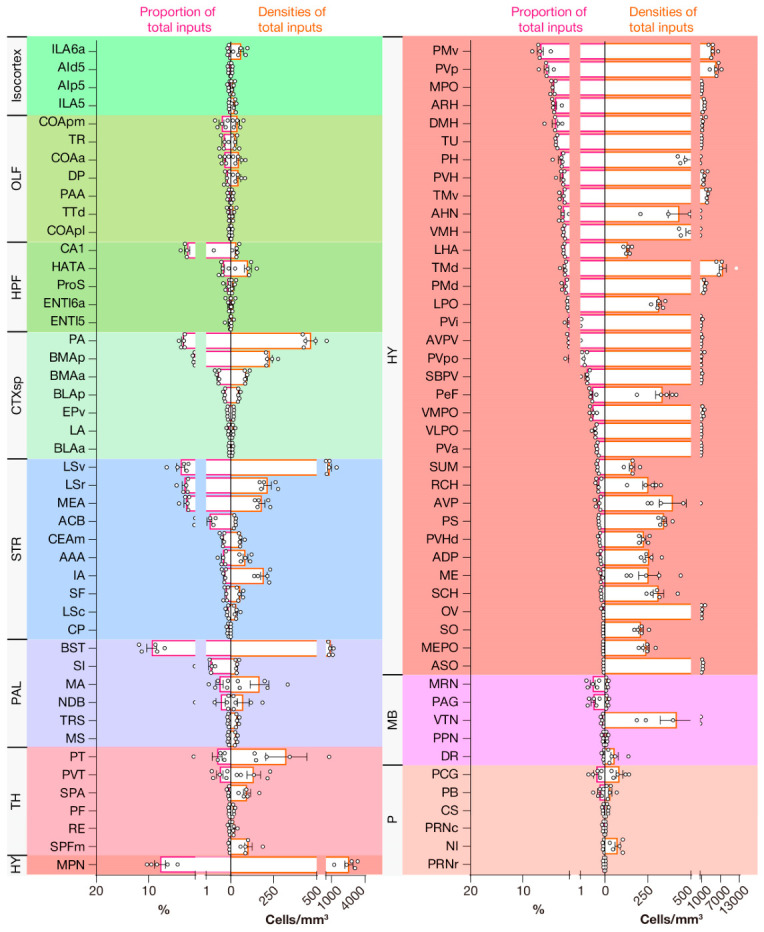
Quantitative mapping of brain-wide monosynaptic inputs to PMv^LepRb^ neurons. Bar graphs detail the proportional contributions of upstream nuclei to the PMv^LepRb^ afferent network. The left *y*-axis displays the fractional weight of each nucleus relative to the total valid inputs (% of total inputs), while the right *y*-axis indicates the cellular innervation density (cells/mm^3^). Minor neural populations constituting <0.05% of the total inputs were excluded from this visualization. Data are shown as mean ± SEM. Open circles denote individual data points (*n* = 5 mice).

**Figure 5:**
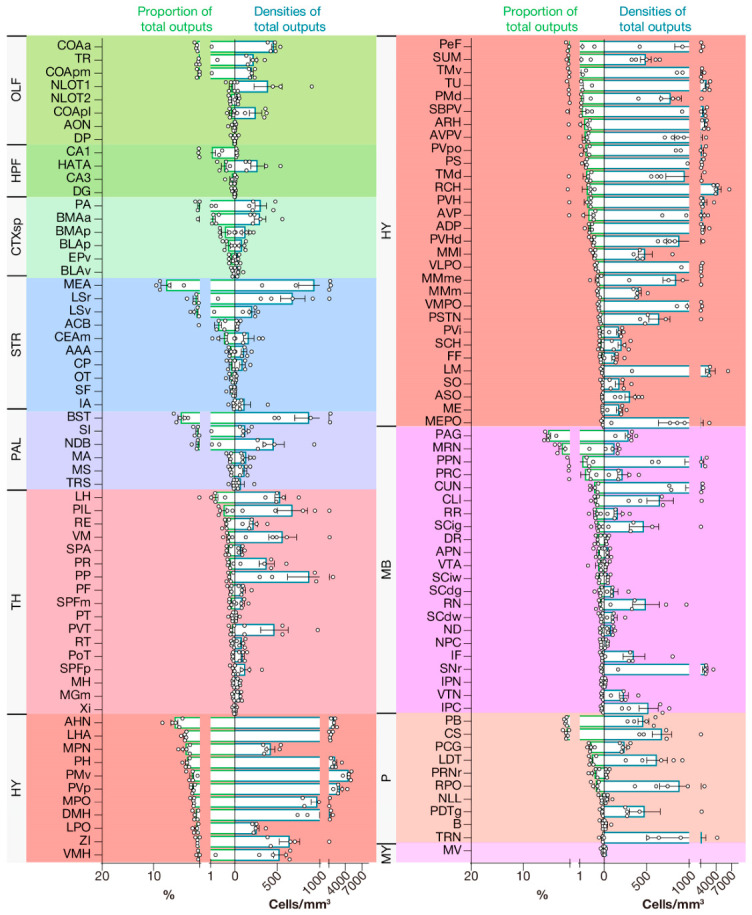
Quantitative mapping of brain-wide monosynaptic outputs from PMv^LepRb^ neurons. Bar graphs detail the proportional distribution of the PMv^LepRb^ efferent network across downstream nuclei. The left *y*-axis displays the fractional weight of each target nucleus relative to the total valid outputs (% of total outputs), while the right *y*-axis indicates the cellular innervation density (cells/mm^3^). Minor downstream target populations constituting <0.05% of the total outputs were excluded from this visualization. Data are shown as mean ± SEM. Open circles denote individual data points (*n* = 5 mice).

To achieve a precise map of the PMv^LepRb^ neural network, we identified a total of 67,135 input neurons and 120,957 output neurons across five mice per viral group. We systematically quantified the proportion and cellular density of trans-synaptically labeled neurons relative to the total brain-wide projections, presenting all nuclei that constituted >0.05% of the overall networks (Figure 4 and Figure 5).

Proportional ranking of the afferent neurons (Figure 4) revealed the BST (9.3%) as the dominant upstream presynaptic node. Furthermore, PMv^LepRb^ neurons receive dense intra-hypothalamic inputs from the MPN (8.6%), local PMv microcircuits (6.9%), PVp (5.1%), ARH (4.2%), MPO (4.1%), DMH (3.6%), TU (3.3%), AHN (2.9%), PH (2.4%), VMH (2.2%), PVH (2.1%), and LHA (2.1%). Significant extra-hypothalamic afferents also originated from striatal nuclei (LSv, 3.8%; LSr, 3.1%), the pallidal MEA (2.8%), the cortical subplate (PA, 3.4%), and the hippocampal CA1 region (2.5%).

Correspondingly, the distribution of efferent targets (Figure 5) exhibited distinct topographies. Sharing similarities with the afferent architecture, dense downstream targets included the pallidal MEA (7.5%), the striatal BST (4.7%), and LSr (1.8%), as well as diverse hypothalamic nuclei (AHN, 5.9%; LHA, 3.9%; MPN, 3.7%; PH, 3.5%; PMv, 2.6%; PVp, 2.4%; MPO, 2.3%; DMH, 1.8%). Notably, diverging from the input network, PMv^LepRb^ neurons established robust, long-range projections to multiple autonomic centers, particularly within the midbrain and pons, including the PAG (5.1%), MRN (2.5%), and PB (1.8%).

**Figure 6:**
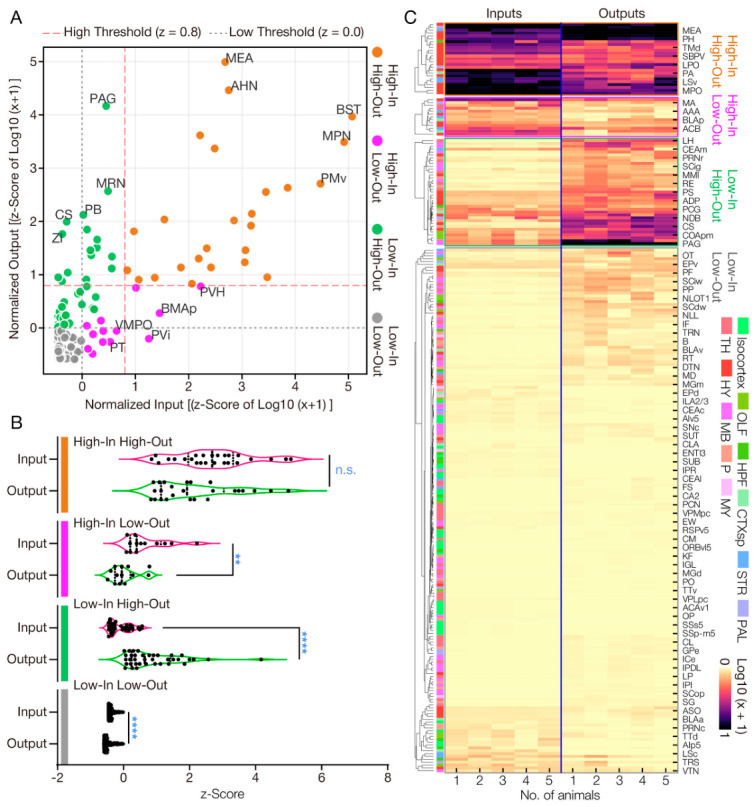
Modular analysis and clustering of PMv^LepRb^ brain-wide connectivity: (**A**) Scatter plot of Z-score standardized input and output connection strengths. Neuronal proportions were standardized via log_10_(x + 1) transformation. Red (Z = 0.8) and gray (Z = 0.0) dashed lines establish the high/low thresholds, highlighting representative core nuclei. (**B**) Violin plots comparing input and output Zscores across four modules (Mann–Whitney *U* test). High-In/High-Out showed no significant difference (*U* = 222, *p* = 0.0807); High-In/Low-Out (*U* = 24, *p* = 0.0012); Low-In/High-Out (*U* = 134, *p* < 0.0001); and Low-In/Low-Out (*U* = 7607, *p* < 0.0001). (**C**) Agglomerative hierarchical clustering heatmap illustrating individual consistency (*n* = 5) and the topological similarity of brain-wide nuclei. The color gradient encodes standardized connection strengths, segregating regional subnetworks based on their distinct projection valences. ** *p* < 0.01, **** *p* < 0.0001, and n.s. = not significant.

To systematically analyze these projection properties, we mapped the fractional distributions of the 176 input and 223 output nuclei using a logarithm transformation, followed by Z-score standardization. This enabled scale-independent comparisons of connectivity strengths across projection directions. Based on these standardized Z-scores (Figure 6A), we precisely segregated the analyzed brain regions into four connectivity-based quadrants using strictly defined high (Z ≥ 0.8, red dashed line) and low (Z < 0.0, gray dashed line) thresholds.

Quantitative analysis indicated that this connectivity distribution is highly non-random. The vast majority of core socio and metabolic hubs clustered in the first quadrant (High-In/High-Out), exhibiting profound interactive reciprocity (e.g., BST: Z_in_ = 5.07, Z_out_ = 3.97; MEA: Z_in_ = 2.69, Z_out_ = 4.99; MPN: Z_in_ = 4.92, Z_out_ = 3.49). The second quadrant (Low-In/High-Out) was enriched with midbrain and pontine nuclei characterized by intense anterograde innervation from the PMv but negligible retrograde feedback. This anatomy anatomically confirms these regions as prominent downstream targets with minimal reciprocal projections (e.g., PAG: Z_in_ = 0.45, Z_out_ = 4.17; MRN: Z_in_ = 0.49, Z_out_ = 2.57; PB: Z_in_ = 0.02, Z_out_ = 2.12). The third quadrant (High-In/Low-Out) contained regions supplying massive inputs to the PMv without serving as primary downstream projection targets (e.g., PVH: Z_in_ = 2.23, Z_out_ = 0.78; BMAp: Z_in_ = 1.46, Z_out_ = 0.28).

**Figure 7:**
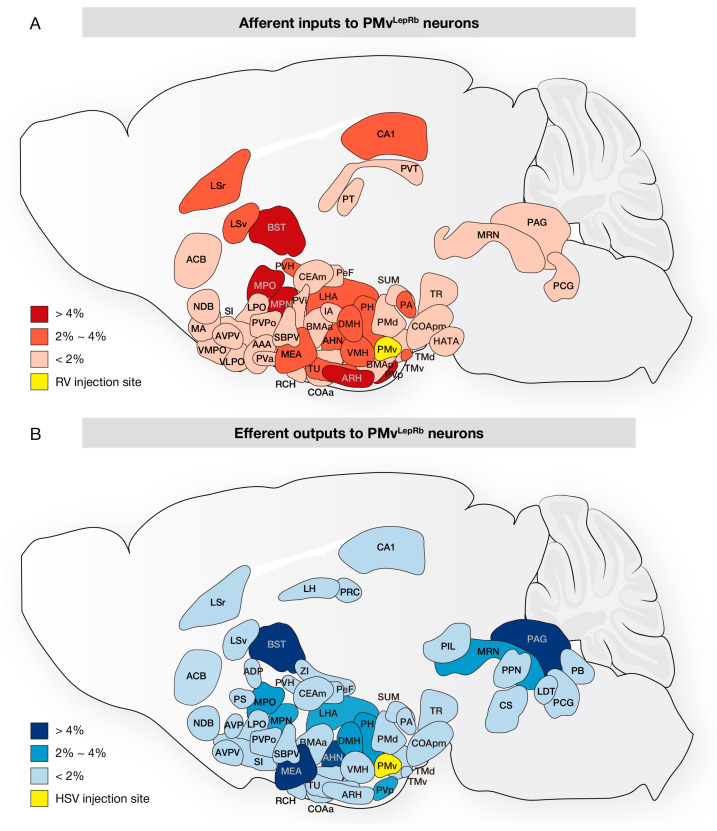
Spatial topography of major input and output nodes in the PMv^LepRb^ connectome. Sagittal brain schematics summarizing the anatomical distribution of the top 50 major afferent (**A**) and efferent (**B**) nuclei. Color gradients—red for inputs and blue for outputs—show the proportion of each region relative to the whole-brain total.

Statistics (Figure 6B) confirmed the divergence of these projections. In the “high-input/high-output” group, there was no significant difference (*ns*) between input and output Z-scores. In contrast, within the asymmetric modules, the statistical results fully matched the scatter plot classification: input values were significantly dominant in the “high-input/low-output” module (*p* < 0.01), whereas output values were markedly higher in the “low-input/high-output” module (*p* < 0.0001), quantitatively validating these regions as specialized downstream targets. The hierarchically clustered heatmap (Figure 6C) demonstrated robust individual consistency across all five animals at both terminals. Finally, to visually contrast these networks, we generated a whole-brain monosynaptic map depicting proportional weights (Figure 7). Color gradients highlighted pronounced regional differences between the telencephalon, diencephalon, and brainstem, providing a solid structural foundation for interpreting the physiological functions of the PMv.

## 4. Discussion

Using Cre-dependent viral tracing techniques (including RV and HSV), we for the first time mapped the whole-brain input and output of PMv^LepRb^ neurons. Our results clearly demonstrate that the role of these neurons extends far beyond relaying basic excitatory output. Instead, they function as a central hub that integrates internal energy reserves and metabolic information, incorporates afferent signals from upstream sensory centers, and projects to downstream regions implicated in innate behaviors. This functional role is enabled by a hierarchical network architecture characterized by prominent asymmetry between its input and output connections.

### 4.1. PMv^LepRb^ Neurons Broadly Integrate Multimodal Information

Canteras and colleagues first mapped the projections of the PMv in 1992 [19]. However, early methods were unable to distinguish LepRb-expressing neurons from other local neuron types. As a result, the precise circuit structure of PMv^LepRb^ neurons remains unclear. Our whole-brain analysis of this afferent network has changed this scenario. We found that the PMv^LepRb^ neurons receive monosynaptic inputs from 176 brain regions, primarily within the hypothalamus and limbic system. When examining the functions of these upstream regions, the input areas exhibit clear functional clustering. This connectivity profile provides an anatomical substrate for the convergence of diverse state signals encompassing metabolism, social behavior, emotion, and reproduction.

The PMv is capable of sensing metabolic status, and our data support this notion. On the one hand, PMv^LepRb^ neurons can be activated by leptin [13]. Meanwhile, as major hypothalamic energy-regulating centers, the ARH, VMH, PVH, and LHA send extensive monosynaptic inputs to PMv^LepRb^ neurons. This arrangement allows for the integration of diverse leptin-sensitive neuronal populations within the hypothalamus. For instance, within the ARH, POMC and AgRP neurons detect hunger levels and control PMv activity via both direct and indirect pathways [9,32], which contributes to the regulation of feeding and energy expenditure. Concurrently, the VMH, PVH, and LHA modulate stress responses [33,34], and their projections to PMv^LepRb^ neurons provide putative anatomical pathways for the integration of such information.

In addition to metabolic processes, the PMv also processes social and environmental information. Our tracing data reveal robust inputs from limbic regions, including the MEA, LS, BST, and CA1 subfield of the hippocampus. The MEA and LS are involved in processing pheromonal signals and innate social behaviors [35,36]. Meanwhile, the BST is primarily involved in regulating anxiety, stress, and motivation [37,38]. Together, these anatomical connections help explain how PMv^LepRb^ neurons integrate complex, instinct-driven behaviors, such as the conflict between social demands and feeding needs.

We identified a prominent structural link between energy balance and reproduction. Reproductive control centers such as the MPO and MPN send direct projections to the PMv [39,40,41]. This positions PMv^LepRb^ neurons as a critical bridge linking internal metabolic state to reproductive drive. This connectivity aligns with classic physiological theory, in which leptin acts on the PMv to regulate GnRH neurons and the broader reproductive axis. We now provide direct connectomic evidence for this organization. By integrating internal metabolic signals and external social cues, PMv^LepRb^ neurons are structurally positioned as a central hypothalamic hub where metabolic, social, and reproductive pathways anatomically converge.

### 4.2. Divergent Projections of PMv^LepRb^ Output Circuits

The output network of PMv^LepRb^ neurons is relatively widely distributed. Among 223 downstream targets, the hypothalamus remains the most prominent output region, underscoring its key role in metabolic regulation. Notably, robust anterograde transsynaptic signals were detected in the midbrain and pons, and almost no reciprocal feedback arises from these hindbrain executive centers. This marked asymmetry suggests a clear functional role; PMv^LepRb^ neurons project predominantly downstream, providing an anatomical framework for potential top-down modulation of specific behavioral and physiological responses in downstream systems.

Within the hypothalamus, numerous PMv^LepRb^ neurons project to the DMH, LHA, PVH, and AHN, providing modulatory feedback. This connectivity suggests an anatomical basis through which these neurons might influence core parameters such as blood glucose levels, body temperature, arousal, and metabolism [42,43]. Outside the hypothalamus, our tracing results reveal strong reciprocal communication between limbic and affective forebrain nuclei, including the BST, MEA, and LS. This implies that the forebrain not only receives external and higher-order cues but also modulates its own excitability via feedback pathways. Together, we provide a direct anatomical map illustrating how leptin influences emotion and motivation [9,44].

When examining downstream brain regions, PMv^LepRb^ neurons send dense projections to brainstem nuclei, including the PAG, MRN, PB, and PPN, with almost no reciprocal input. These regions represent critical centers for regulating autonomic behaviors. They control autonomic output, manage acute stress, and trigger innate survival strategies such as escape or defensive behaviors [45,46]. For instance, the PAG and PB govern nociceptive responses and cardiopulmonary rhythms [47,48,49]. These projections to the hindbrain provide an anatomical substrate for how metabolic shifts can modulate stress responses and survival instincts. Our modularity analysis fully supports this organization: the “low-input/high-output” category comprises 37 regions, nearly all of which are brainstem nuclei.

### 4.3. The Role of the PMv^LepRb^ Neural Network in Male-Specific Functions

Male social competition drains energy and carries serious survival risks. Whether defending territory, securing resources, or fighting for mates, animals must continuously weigh internal energy reserves against external pressures. Our neural circuit map reveals that PMv^LepRb^ neurons serve as a structural node located at the intersection of circuits associated with this delicate balance.

Connections between PMv^LepRb^ neurons, the VMH, and the MEA form the anatomical basis for the PMv to process inter-male social signals. When entering a competitive context, males detect pheromonal signals—such as those captured by the Vmn2r53 receptor and relayed to the PMv via the vomeronasal system [24,50]. This male-specific information flow reaches the PMv, which in turn provides dense projections to the VMH, whose ventrolateral subdivision acts as an aggression center capable of triggering acute aggressive responses [51]. Our newly mapped long-range projections to the brainstem act in concert with this process. Direct projections from PMv^LepRb^ neurons to the PAG constitute a putative anatomical route that may be involved in postural adjustments [48] and hierarchical social relationships among animals [52]. Intense fighting increases the body’s demand for oxygen and waste clearance. We identified dense pathways from the PMv to the PB that provide a direct route for rapid autonomic nervous system mobilization [53,54]. At the onset of fighting, the dense PMv projections to the PB offer an anatomical pathway that could potentially support cardiopulmonary modulation, ensuring adequate circulatory and respiratory support during this high-intensity physical activity.

Combining this neural tracing map with the role of the PMv in sensing leptin signals reveals the functional foundation of this system. When energy reserves are sufficient, leptin exerts a permissive effect, allowing the initiation of corresponding energy-consuming behaviors such as territorial defense and courtship competition, ultimately enhancing reproductive success. Under conditions of food scarcity and depleted energy, the PMv reduces excitability, prompting animals to forgo nonessential fighting and courtship to avoid fatal energy collapse.

### 4.4. Study Limitations and Future Perspectives

Our study provides a comprehensive tracing atlas. However, a gap still exists between anatomical connections and actual physiological function. Viral tracing can demonstrate morphological connections between neurons, but it cannot reveal whether these synapses are functionally connected. To address this question, optogenetics must be combined with real-time electrophysiological recordings. This approach will ultimately uncover the true electrophysiological properties and functional strength of these core pathways.

A static connectivity map alone cannot establish causality. Identifying a structural pathway does not necessarily confirm that it drives a specific behavior. To confirm the precise role of particular projections under defined conditions—for instance, whether the PMv-to-PAG pathway is attenuated under leptin system dysfunction, reducing social dominance in animals, more detailed and comprehensive studies will be required.

Future research must further explore functional and clinical implications. A notable limitation of the present study is the exclusive use of male mice. Therefore, it is critical for future studies to systematically map the PMv^LepRb^ connectome in females to elucidate sex-dependent differences in anatomical circuitry, functional dynamics, and neurochemical phenotypes. Additionally, we need to investigate specific genetic subpopulations of PMv^LepRb^ neurons. For example, do neurons projecting to the midbrain and pons represent distinct subpopulations from those providing feedback outputs to the hypothalamus? We also need to examine changes in the anatomical and electrophysiological properties of these synapses under metabolic conditions, such as obesity or diabetes. This may open new research paradigms, offering a novel perspective for investigating complex metabolic disorders and psycho-behavioral abnormalities based on this precisely defined network.

## 5. Conclusions

Our study presents the first comprehensive, whole-brain input–output atlas of PMv^LepRb^ neurons. Our data reveal that these neurons integrate diverse inputs encoding metabolic, social, emotional, and reproductive states; in turn, they both exert feedback modulation on their upstream afferent regions and send robust excitatory efferent projections to the brainstem. This atlas provides a critical anatomical substrate for delineating how the brain couples internal metabolic state to survival-related innate behaviors.

## Data Availability

The data presented in this study are available on request from the corresponding author. The raw immunofluorescence data are not publicly available due to their extremely large file sizes and the lack of a suitable public repository for hosting them.

## Abbreviations

The following abbreviations are used in this manuscript:

LepRb	Leptin receptor b
RV	Rabies virus
HSV	Herpes simplex virus
BSL-2	Biosafety Level 2
CCFv3	Common Coordinate Framework version 3
PMv	Ventral premammillary nucleus
MPO	Medial preoptic area
MPN	Medial preoptic nucleus
VMH	Ventromedial hypothalamic nucleus
DMH	Dorsomedial hypothalamic nucleus
ARH	Arcuate hypothalamic nucleus
LHA	Lateral hypothalamic area
PVH	Paraventricular hypothalamic nucleus
BST	Bed nuclei of the stria terminalis
MEA	Medial amygdalar nucleus
LSr/LSv	Lateral septal nucleus, rostral (ventral) part
PAG	Periaqueductal gray
PB	Parabrachial nucleus
MRN	Midbrain reticular nucleus
